# Asymptomatic Lymphogranuloma Venereum in Men who Have Sex with Men, United Kingdom

**DOI:** 10.3201/eid2201.141867

**Published:** 2016-01

**Authors:** Cara Saxon, Gwenda Hughes, Catherine Ison, for the UK LGV Case-Finding Group

**Affiliations:** Author affiliation: Public Health England, London, UK

**Keywords:** Lymphogranuloma venereum, *Chlamydia trachomatis*, bacteria, sexually transmitted infections, sexually transmitted diseases, men who have sex with men, bacterial screening, asymptomatic, United Kingdom, serovars, MSM, LGV, CT, STI, STD

## Abstract

We investigated prevalence of lymphogranuloma venereum (LGV) among men who have sex with men who were tested for chlamydia at 12 clinics in the United Kingdom during 10 weeks in 2012. Of 713 men positive for *Chlamydia trachomatis*, 66 (9%) had LGV serovars; 15 (27%) of 55 for whom data were available were asymptomatic.

Lymphogranuloma venereum (LGV) is a sexually transmitted infection (STI) caused by the L1, L2, and L3 serovars of *Chlamydia trachomatis* (CT). An LGV outbreak among men who have sex with men (MSM) first reported in the Netherlands in 2003 has since spread across other industrialized countries (*1*). Cases are typically seen among white, HIV-positive MSM who report unprotected anal intercourse, other high-risk behaviors, and STI co-infection and who commonly have symptoms of proctitis (i.e., rectal pain, rectal discharge, bloody stools, constipation, and tenesmus) (*2*).

The United Kingdom now has the largest documented outbreak of LGV among MSM worldwide (*3**,**4*). Infection control in England has relied on CT DNA typing and treatment of symptomatic MSM who have CT-positive rectal infections and their contacts, as well as health promotion. These measures were supported by a large prospective study in the United Kingdom during 2006–2007 that reported <6% of LGV CT infections were asymptomatic (*5*). However, studies in the Netherlands and Germany, and a smaller UK study, have reported higher proportions (17%–53%) of asymptomatic infection (*6*–*8*). We reinvestigated the prevalence of asymptomatic LGV CT infection among MSM in the United Kingdom to assess whether it may be sustaining the current epidemic.

## The Study

In the UK, STI clinics are open access and provide free testing and treatment. Regular STI and HIV screening is encouraged for sexually active MSM with or without symptoms (*9*). A full medical and sexual history are recorded for all patients, and a physical examination is done for those with symptoms.

Twelve UK STI clinics participated; all serve cities with large MSM populations and routinely screen MSM for CT by examining urine or swab samples of the pharynx, urethra, and rectum (either clinician-obtained or self-taken) according to UK guidelines (*10*). All MSM tested for CT during September 24–December 7, 2012, were included except those who had received antibiotic drugs during the previous 6 weeks. 

More than 10,000 CT tests were performed during the study period. Local laboratories performed routine testing for CT and referred all positive specimens from study participants to the Sexually Transmitted Bacteria Reference Unit of the national reference laboratory in London to test for LGV. At the reference laboratory, all specimens underwent extraction by using the Roche MagNA Pure LC extractor (Roche Diagnostics, Indianapolis, IN, USA), then CT confirmation by using a plasmid targeted real-time PCR and an LGV-specific real-time PCR targeting the *pmpH* deletion on the RotorGene (QIAGEN, Valencia, CA, USA). Details of the LGV reference service were previously published (*11*).

Clinical data for symptoms were submitted for all study participants to Public Health England (PHE) through a secure web portal (Technical Appendix Figure). Patients reporting symptoms at first medical examination or follow-up were defined as symptomatic. Those with no symptoms at first examination or follow-up were defined as asymptomatic. Additional clinical data were available from the national anonymized patient-level electronic surveillance system (the Genitourinary Medicine Clinic Activity Dataset [GUMCADv2]), which records all tests and diagnoses in STI clinics in England (*12*).

PHE has authority to collect anonymized patient-level data for public health monitoring and infection control. The study was reviewed in PHE’s research and development office and deemed to fit this criterion.

We used univariable and multivariable logistic regression modeling in STATA version 13.1 (StataCorp LP, College Station, TX, USA) to investigate risk factors associated with LGV versus non-LGV CT infection and asymptomatic versus symptomatic LGV. A clinic in Glasgow, Scotland, was excluded from risk factor analyses because it does not report GUMCADv2 (Figure).

**Figure:**
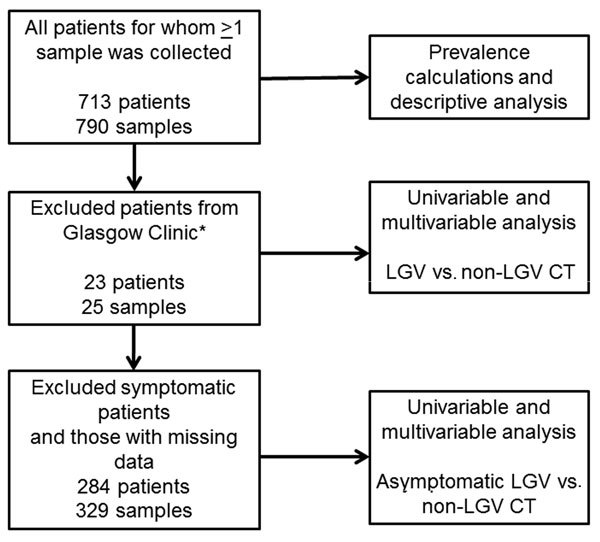
Data analysis flowchart for univariable and multivariable analyses of symptomatic lymphogranuloma venereum (LGV) versus non-LGV *Chlamydia trachomatis* (CT) infection (Table 1) and asymptomatic LGV versus non-LGV CTinfection (Table 2) in men who have sex with men, United Kingdom*Patients from Glasgow were excluded from risk factor analyses because they do not routinely report to the Genitourinary Medicine Clinic Activity Dataset.

During the study period, 921 eligible specimens were received for DNA typing. On confirmatory testing, 90 (10%) specimens were CT negative, 36 (4%) inhibitory, and 1 (0.1%) equivocal; 4 (0.4%) were not tested. CT infection was confirmed in 790 specimens from 713 patients; these specimens then underwent DNA typing. Overall, we found 69 (9%) LGV CT–positive specimens from 66 (9%) patients and 721 (91%) non-LGV CT–positive specimens from 647 (91%) patients (Table 1). Co-infection with LGV CT at 1 anatomic site and non-LGV CT at a different site was found in 4/713 (0.6%) patients. Clinical data coordinating with the symptom checklist and GUMCADv2 were available for 95% (680/713) and 87% (603/690) of CT-positive patients, respectively. During the study period, GUMCADv2 recorded 1,097 CT diagnoses among 10,143 MSM screened at the 11 STI clinics in England, showing an estimated CT prevalence of 10.8%.

**Table 1:** Descriptive, univariable, and multivariable analysis of patients diagnosed with symptomatic versus nonsymptomatic LGV CT infection among men who have sex with men, by demographic and behavioral characteristics, United Kingdom*

Patient characteristics	No. (%) or median [IQR]				Univariable analysis			Multivariable analysis†		
	All CT	Non-LGV CT	LGV CT		OR (95% CI)	p value		OR (95% CI)		p value
All patients	713 (100)	647 (90.7)	66 (9.3)		ND	ND		ND		ND
Clinic location, n = 713										
London	563 (79.0)	512 (79.1)	51 (77.3)		1	0.73				
Manchester	85 (11.9)	75 (11.6)	10 (15.2)		1.34 (0.65–2.75)					
Brighton	42 (5.9)	38 (5.9)	4 (6.1)		1.06 (0.36–3.08)					
Glasgow‡	23 (3.2)	22 (3.4)	1 (1.5)							
Infection site, n = 710										
Nonrectal	221 (31.1)	217 (33.7)	4 (6.1)		1			1		
Rectal	489 (68.9)	427 (66.3)	62 (93.9)		7.83 (2.81–21.82)	<0.001		10.08 (3.37–30.17)		<0.001
Multiple infection sites, n = 713										
No	641 (89.9)	581 (89.8)	60 (90.9)		1					
Yes	72 (10.1)	66 (10.2)	6 (9.1)		0.89 (0.37–2.15)	0.8				
Symptoms present, n = 650										
No	453 (69.7)	438 (73.6)	15 (27.3)		1			1		
Yes	197 (30.3)	157 (26.4)	40 (72.7)		7.93 (4.20–14.99)	<0.001		13.33 (6.53–27.21)		<0.001
Age, y, n = 710	33 [27–42]	33 [27–42]	39 [33–46]							
18–24	108 (15.2)	106 (16.5)	2 (3.0)		1	0.002				
25–34	276 (38.9)	258 (40.1)	18 (27.3)		3.72 (0.85–16.31)					
35–44	192 (27.0)	166 (25.8)	26 (39.4)		7.92 (1.84–34.15)					
>44	134 (18.9)	114 (17.7)	20 (30.3)		9.11 (2.08–39.93)					
Ethnicity, n = 603										
White	480 (79.6)	432 (79.3)	48 (82.8)		1	0.17				
Black or Black British	28 (4.6)	27 (5)	1 (1.7)		0.33 (0.44–2.51)					
Mixed	27 (4.5)	27 (5)	0		NP					
Asian and Asian British	21 (3.5)	21 (3.9)	0		NP					
Other ethnic groups	23 (3.8)	19 (3.5)	4 (6.9)		1.89 (0.62–5.80)					
Unknown	24 (4)	19 (3.5)	5 (8.6)		2.37 (0.85–6.63)					
HIV status, n = 603										
Negative	367 (60.9)	350 (64.2)	17 (29.3)		1			1		
Positive	236 (39.1)	195 (35.8)	41 (70.7)		4.33 (2.40–7.82)	<0.001		3.63 (1.80–7.32)		<0.001
No. sexual partners in previous 3 mo, n = 635										
No. partners	3 [1–5]	3 [1–5]	3 [1–8]							
0–1	169 (26.6)	153 (26.3)	16 (29.6)		1	0.26				
2–5	321 (50.6)	299 (51.5)	22 (40.7)		0.66 (0.34–1.31)					
>6	145 (22.8)	129 (22.2)	16 (29.6)		1.13 (0.54–2.36)					
Concurrent sexually transmitted infection, n = 631										
No	451 (71.5)	406 (71)	45 (76.3)		1					
Yes	180 (28.5)	166 (29)	14 (23.7)		0.76 (0.41–1.42)	0.39				
STI within previous 12 mo, n = 594										
No	469 (79)	435 (80.3)	34 (65.4)		1					
Yes	125 (21)	107 (19.7)	18 (34.6)		2.15 (1.17–3.96)	0.01				

*n values indicate number of patients for which data were available in each category. CT, *Chlamydia trachomatis*; LGV, lymphogranuloma venereum; IQR, interquartile range; OR, odds ratio; ND, no data were available; NP, not performed. Blank cells indicate not applicable. †Multivariable analysis adjusted for age and location. ‡Glasgow patients were excluded from all univariable and multivariable analyses because they do not routinely report to the Genitourinary Medicine Clinic Activity Dataset.

Compared to those positive for non-LGV CT, patients with LGV CT infection were older and more likely to be symptomatic, to be HIV-positive, to have rectal infection, and to have had a previous STI diagnosis. In adjusted logistic regression analysis, symptomatic infection (adjusted odds ratio [aOR] 13.33; p<0.001), rectal infection (aOR 10.08; p<0.001) and being HIV-positive (aOR 3.63; p<0.001) remained statistically significant (Table 1).

Of those with LGV for whom data were available, 27% (15/55) overall and 22% (12/54) with rectal-only infection were asymptomatic. Study prevalence of asymptomatic LGV was 2.3% (15/650) overall and 3.8% (9/236) in HIV-positive MSM (Tables 1, 2).

**Table 2:** Descriptive, univariable and multivariable analysis of patients in whom asymptomatic LGV CT or asymptomatic non-LGV CT infection was diagnosed, by demographic and behavioral characteristics, United Kingdom*

Patient characteristics	No. (%) or median [IQR]								
	All asymptomatic CT	Asymptomatic non-LGV CT	Asymptomatic LGV		Univariable analysis			Multivariable analysis†	
					OR (95% CI)	p value		OR (95% CI)	p value
All patients	429 (100.0)	414 (96.5)	15 (3.5)				
Clinic location, n = 429									
London	333 (77.6)	323 (78.0)	10 (66.7)		1	0.47			
Manchester	54 (12.6)	52 (12.6)	2 (13.3)		1.24 (0.26–5.83)				
Brighton	26 (6.1)	24 (5.8)	2 (13.3)		2.69 (0.56–12.99)				
Glasgow‡	16 (3.7)	15 (3.6)	1 (6.7)						
Infection site, n = 427									
Nonrectal	129 (30.2)	126 (30.6)	3 (20.0)		1				
Rectal	298 (69.8)	286 (69.4)	12 (80.0)		1.67 (0.46–6.08)	0.44			
Multiple infection sites, n = 429									
No	397 (92.5)	383 (92.5)	14 (93.3)		1				
Yes	32 (7.5)	31 (7.5)	1 (6.7)		0.95 (0.12–7.48)	0.96			
Age, y, n = 429	33 [27–42]	33 [26–42]	38 [29–44]	­					
18–24	73 (17.0)	72 (17.4)	1 (6.7)		1	0.64			
25–34	172 (40.1)	167 (40.3)	5 (33.3)		2.19 (0.25–19.07)				
35–44	105 (24.5)	99 (23.9)	6 (40.0)		3.72 (0.43–32.59)				
>44	79 (18.4)	76 (18.4)	3 (20.0)		2.8 (0.28–27.55)				
Ethnicity, n = 378									
White	302 (79.9)	290 (79.5)	12 (92.3)		1	0.32			
Black or Black British	18 (4.8)	18 (4.9)	0		NP				
Mixed	19 (5.0)	19 (5.2)	0		NP				
Asian and Asian British	17 (4.5)	17 (4.7)	0		NP				
Other ethnic groups	13 (3.4)	13 (3.6)	0		NP				
Unknown	9 (2.4)	8 (2.2)	1 (7.7)		3.02 (0.35–26.13)				
HIV status, n = 378									
Negative	235 (62.2)	231 (63.3)	4 (30.8)		1				
Positive	143 (37.8)	134 (36.7)	9 (69.2)		3.88 (1.17–12.84)	0.03		3.91 (0.92–16.66)	0.06
No. sexual partners in previous 3 mo, n = 401									
No. partners	3 [1–5]	3 [1–5]	3 [2–5]						
0–1	106 (26.4)	103 (26.7)	3 (20.0)		1	0.87			
2–5	207 (51.6)	198 (51.3)	9 (60.0)		1.41 (0.37–5.44)				
>6	88 (21.9)	85 (22.0)	3 (20.0)		1.16 (0.23–5.90)				
Concurrent sexually transmitted infections, n = 398									
No	295 (74.1)	283 (73.7)	12 (85.7)		1				
Yes	103 (25.9)	101 (26.3)	2 (14.3)		0.47 (0.10–2.12)	0.32			
STI during previous 12 mo, n = 374									
No	297 (79.4)	291 (80.4)	6 (50.0)		1				
Yes	77 (20.6)	71 (19.6)	6 (50.0)		4.1 (1.28–13.09)	0.02		3.1 (0.87–10.99)	0.08

*n values indicate number of patients for which data were available in each category. CT, *Chlamydia* *trachomatis*; LGV, lymphogranuloma venereum; No., number; IQR, interquartile range; CI, confidence interval; OR, odds ratio; NP, not performed. Blank cells indicate not applicable. †Multivariable analysis adjusted for age and location ‡Glasgow patients were excluded from all univariable and multivariable analyses because they do not routinely report to the Genitourinary Medicine Clinic Activity Dataset.

Of the 15 patients with asymptomatic LGV, 12 (80%) had rectal, 2 (13%) urethral, and 1 (7%) pharyngeal infections. All cases of asymptomatic LGV were from patients with single-site infection.

Among asymptomatic patients, those with LGV were more likely to be HIV-positive (69% vs. 31%; odds ratio 3.88; p = 0.03) and to have had an STI in the past 12 months (50% vs. 20%; odds ratio 4.1; p = 0.02) than those infected with non-LGV CT. These characteristics were only weakly associated in the adjusted analysis (aOR 3.91, p = 0.06, and aOR 3.1, p = 0.08, respectively).

## Conclusions

This large multicenter case-finding study found a higher rate of asymptomatic LGV (27%) than previously reported in the United Kingdom, in agreement with studies done in Germany and the Netherlands. LGV case-patients were typically older, white, HIV-positive MSM who had a concurrent or recent STI diagnosis. Most infections were rectal; few urethral and pharyngeal infections were detected.

The number of CT infections confirmed at the reference laboratory (713) was lower than those reported to national surveillance (1,097), possibly related to differences in test sensitivity, degradation of CT DNA during transportation, or incorrect surveillance coding. No patients were excluded because of study restrictions; therefore, it is likely the study case-patients were representative of all MSM with diagnoses of CT infection in the United Kingdom.

More than one quarter of LGV cases in the United Kingdom may go undiagnosed if those who have asymptomatic chlamydial infection are not tested, as is the current strategy. Recommending that all CT-positive specimens from MSM be DNA tested for LGV serovars is unlikely to be cost-effective or feasible. However, because 3.8% of asymptomatic HIV-positive MSM had LGV (i.e., in excess of the recommended 3% prevalence threshold for CT screening [*13*]), inclusion of these patients in the testing algorithm, as is done in Scotland (*14*)*,* may be warranted.

Whether LGV symptomatology in the United Kingdom has changed or asymptomatic cases were previously missed is unclear. Changes in screening practice or selection pressure for asymptomatic infection after treatment of persons with symptomatic infection may have contributed. Most asymptomatic patients will be treated for non-LGV CT infection, but if treatment is suboptimal, it may not prevent onward transmission (*15*)*.*

An undiagnosed reservoir of CT infection is unlikely to be the sole cause of the current epidemic. High-risk sexual behavior remains a substantive challenge for control of LGV and related epidemics among MSM (*3*). Future public health strategies will require a combined strategy of increased testing, prompt treatment, and continued promotion of safer sexual behavior among MSM.

**Technical Appendix.** Symptom checklist posted on Public Health England site for detection of asymptomatic lymphogranuloma venereum infection in men who have sex with men.
