# Supplementary material for: Asymptomatic Lymphogranuloma Venereum in Men who Have Sex with Men, United Kingdom
Source: Emerg Infect Dis. 2016 Jan;22(1):112–6. doi: 10.3201/eid2201.141867 (PMC4696683; doi:10.3201/eid2201.141867)
Supplement: Supplementary file 1 — Technical Appendix. Symptom checklist posted on Public Health England site for detection of asymptomatic lymphogranuloma venereum infection in men who have sex with men. [file 14-1867-Techapp-s1.pdf]

Article DOI: http://dx.doi.org.10.3201/eid2112.141867

Asymptomatic Lymphogranuloma Venereum in Men who Have Sex with Men, United Kingdom

Technical Appendix

Checklist of symptoms for all patients CT-positive

**Patient details**					
**Patient ID:**		**Date of birth:** _ _ / _ _ / _ _ _ _			
**Sexual history**					
**Last sexual intercourse**	Date: _ _ / _ _ / _ _ _ _	**or**	# of weeks ago: _	**or**	# of months ago: _ _
**# of sexual partners last 6 months:** _ _					
**Attendance history**					
**1st attendance date:** _ _ / _ _ / _ _ _ _	**2nd attendance date:** _ _ / _ _ / _ _ _ _	**DNA at 2nd attendance:** Yes / No / Don't know			
**Diagnosis & treatment**					

**Sample**	Date: _ _ / _ _ / _ _ _ _	**Urethral**	**Rectal**	**Pharyngeal**
	**Chlamydia positive** *	Yes / No / Don't know	Yes / No / Don't know	Yes / No / Don't know
	**LGV positive** *	Yes / No / Don't know	Yes / No / Don't know	Yes / No / Don't know
**Was patient treated for chlamydia between 1st and 2nd attendance?** Yes / No / Don't Know				
**Symptoms** (* *please provide answers for ALL symptom indicators below (white and grey) for LGV positives and just those in white for chlamydia positives/LGV negatives)*				
**Symptoms at 1st attendance:** Yes / No / Don't know		**Symptoms at 2nd attendance:** Yes / No / Don't know		
**Onset of symptoms**	Date: _ _ / _ _ / _ _ _ _	**or** # of weeks ago: _	**or** # of months ago: _ _	

* **Chlamydia & LGV symptoms** *(please answer for ALL patients)*			**Duration of symptoms (days)**
**Rectal**	Tenesmus / hard	Yes / No / Don't know	_ _
	Anorectal pain / hard	Yes / No / Don't know	_ _
	Rectal bleeding / hard	Yes / No / Don't know	_ _
	Rectal Discharge / hard	Yes / No / Don't know	_ _
**Penile / urethral**	Penile discharge / hard	Yes / No / Don't know	_ _
	Epididymitis / soft	Yes / No / Don't know	_ _
	Pain on urination / soft	Yes / No / Don't know	_ _
**Pharyngeal**	Pharyngitis / soft	Yes / No / Don't know	_ _
	Tonsillitis / soft	Yes / No / Don't know	_ _
**Other**	Lymphadenopathy, (please specify site in 'Other')	Yes / No / Don't know	_ _
	Swollen / painful joints	Yes / No / Don't know	_ _
	Itchy / red eyes	Yes / No / Don't know	_ _
	Other (please specify)		_ _
* **Additional LGV symptoms** *(please answer for LGV patients ONLY)*			**Duration of symptoms (days)**
**Rectal**	Anorectal ulceration / hard	Yes / No / Don't know	_ _
	Bloody stools / hard	Yes / No / Don't know	_ _
	Contact bleeding / hard	Yes / No / Don't know	_ _
	Recent constipation / hard	Yes / No / Don't know	_ _
	Change in bowel habit / soft	Yes / No / Don't know	_ _
	Perianal itch / irritation / soft	Yes / No / Don't know	_ _
**Penile / urethral**	Penile ulcers / abscess / hard	Yes / No / Don't know	_ _
**Pharyngeal**	Swollen/painful neck nodes / hard	Yes / No / Don't know	_ _
	Mouth ulcers / soft	Yes / No / Don't know	_ _
**Other**	Swollen inguinal lymph nodes/buboes / hard	Yes / No / Don't know	_ _
	Painful lymph nodes / hard	Yes / No / Don't know	_ _
	Erythema nodosum (tender red nodules on shins) / hard	Yes / No / Don't know	_ _
	Weight loss / soft	Yes / No / Don't know	_ _
	Fever / soft	Yes / No / Don't know	_ _
	Muscle / joint pain / soft	Yes / No / Don't know	_ _
	Malaise / soft	Yes / No / Don't know	_ _
	Other (please specify)		_ _

Technical Appendix Figure. Online symptom checklist posted on Public Health England site for detection of asymptomatic lymphogranuloma venereum infection in men who have sex with men, United Kingdom.
